# Adjusting Iron Markers for Inflammation Reduces Misclassification of Iron Deficiency After Total Hip Arthroplasty

**DOI:** 10.3390/jcm15010259

**Published:** 2025-12-29

**Authors:** Alexander Tham, Donald C. McMillan, Dinesh Talwar, Stephen T. McSorley

**Affiliations:** 1 Academic Unit of Surgery, School of Medicine, University of Glasgow, Glasgow Royal Infirmary, Glasgow G31 2ER, UK; 2 Department of Orthopaedic Surgery, New York University Langone Health, New York, NY 10022, USA; 3 The Scottish Trace Elements and Micronutrients Reference Laboratory, Department of Clinical Biochemistry, Glasgow Royal Infirmary, Glasgow G4 0SF, UK

**Keywords:** C-reactive protein, systemic inflammatory response, iron status, iron deficiency, surgery

## Abstract

**Background:** Preoperative anemia is common among patients undergoing arthroplasty and is associated with increased transfusion requirements and worse outcomes. Current perioperative pathways rely on iron studies to guide intravenous iron supplementation, but systemic inflammation triggered by surgery profoundly alters iron markers, risking misclassification of iron deficiency. This study evaluated whether adjusting iron indices for inflammatory markers improves diagnostic accuracy after total hip arthroplasty (THA). **Methods:** In this prospective cohort study, 20 patients undergoing elective primary THA at a single center were enrolled. Patients with preoperative inflammation were excluded. Serum iron, transferrin, transferrin saturation (TSAT), CRP, and albumin were measured preoperatively and on postoperative days (PODs) 1, 2, 3, and 90. Serum iron was adjusted for systemic inflammation using a validated regression equation incorporating CRP and albumin, and adjusted TSAT was calculated accordingly. Absolute iron deficiency was defined as serum iron < 10 µmol/L, and functional iron deficiency was defined as TSAT < 20%. Comparisons were made using Wilcoxon’s signed-rank test and ANOVA. **Results:** In the 20 included patients, a pronounced systemic inflammatory response was observed, with CRP peaking on POD 2 (median, 162 mg/L) and albumin falling to 32 g/L on POD 1 (both *p* < 0.001). Unadjusted serum iron and TSAT fell sharply, with nearly all patients classified as iron-deficient in the first three postoperative days. Adjustment for CRP and albumin significantly attenuated these declines: on POD 2, median iron was 8.2 µmol/L adjusted versus 2.0 µmol/L unadjusted (*p* < 0.001), and TSAT was 19% versus 4% (*p* < 0.001). Misclassification of iron deficiency fell by 40–50% with adjustment, and by POD 90, adjusted indices approximated baseline values. **Conclusions**: Systemic inflammation after THA markedly suppresses iron indices, leading to widespread misclassification of iron deficiency. Adjustment for CRP and albumin reduces this misclassification and provides a more accurate assessment of perioperative iron status. These findings complement existing evidence supporting intravenous iron supplementation by highlighting a diagnostic refinement that could improve patient selection for therapy.

## 1. Introduction

Preoperative anemia is a common finding in patients undergoing elective orthopedic surgery, with reported prevalence ranging from 20% to 40% [1,2,3]. Its presence is associated with increased perioperative morbidity [4,5], higher transfusion requirements [2], and up to a fivefold increase in 90-day postoperative mortality [6]. Major joint arthroplasty procedures, such as total hip arthroplasty (THA), utilize a substantial proportion of blood transfusion resources, as intraoperative blood loss may approach one-third of total body volume [7]. Total hip arthroplasty is among the most performed elective surgical procedures globally, with annual case volumes continuing to rise and projected to increase substantially over the coming decades [8,9,10].

For these reasons, accurate diagnosis and management of anemia have become essential elements of perioperative optimization strategies designed to improve outcomes and reduce transfusion burden [2,7,11]. Current pathways rely heavily on iron studies to guide treatment decisions, including the use of intravenous iron supplementation.

However, the physiological and biochemical effects of the underlying diseases and of the surgery itself are not always fully considered in perioperative anemia management and may substantially affect the interpretation of iron indices [12,13].

Iron deficiency anemia, characterized by hypochromic microcytic indices, accounts for approximately 20–30% of anemia in elective surgical patients [14]. In contrast, a larger proportion of patients present with normocytic anemia, which has been shown to be more than twice as prevalent in both major elective orthopedic surgery [15] and colorectal cancer patients [16]. Normocytic anemia is frequently associated with elevated markers of systemic inflammation [16]. In this setting, inflammatory cytokines stimulate hepatic hepcidin production, leading to sequestration of iron within the reticuloendothelial system and reduced availability of circulating iron for erythropoiesis, despite preserved or elevated iron stores [17,18,19,20]. This state is referred to as functional iron deficiency and represents a key feature of anemia of chronic disease or inflammation. In such cases, transferrin saturation (TSAT) below 20% is widely used to identify functional iron deficiency in the presence of normal ferritin and red-cell indices [18]. Importantly, inflammation may also directly suppress erythropoiesis within the bone marrow, impair erythropoietin signaling, and reduce erythrocyte lifespan, thereby further contributing to perioperative anemia.

The assessment of iron status in the perioperative period is further complicated by the systemic inflammatory response triggered by surgical trauma. Acute-phase reactants such as C-reactive protein (CRP) and ferritin increase in concentration following surgery, whereas negative-phase proteins, including albumin and transferrin, decrease in concentration [17,18]. Serum iron and TSAT levels, which are markers commonly used to guide iron therapy, are suppressed in the early postoperative period. This inflammatory suppression may lead to overdiagnosis of iron deficiency and inappropriate perioperative iron supplementation [18,21]. As a result, the true prevalence of iron deficiency may be lower when effects of systemic inflammation are appropriately taken into account.

Several approaches have been proposed to adjust iron markers for inflammation. The Biomarkers Reflecting Inflammation and Nutritional Determinants of Anemia (BRINDA) project developed regression-based correction methods using CRP and α-1-acid glycoprotein (AGP) to account for the effects of inflammation on iron indices [22]. However, AGP is not routinely measured in perioperative practice, thereby limiting its clinical utility. Our group has previously developed a regression equation to correct serum iron for inflammation using CRP and albumin. These are two widely available biomarkers in the perioperative setting that have demonstrated utility in non-orthopedic cohorts [18,21]. Given the increasing role of perioperative iron supplementation in arthroplasty and the recent evidence supporting its efficacy [23], accurate diagnostic tools are needed to ensure therapy is appropriately targeted.

Additionally, inflammation can cause direct suppression of erythropoiesis within the marrow, indirect suppression via inhibition of erythropoietin, and reduction in the circulating erythrocyte lifespan, leading to a picture more in keeping with the anemia of chronic disease/anemia of inflammation [12]. In these cases, transferrin saturation (TSAT) less than 20% is considered to diagnose functional iron deficiency in the presence of normal ferritin and red-cell measurements [18]. Transferrin, ferritin, and serum iron are all affected during the evolution of the systemic inflammatory response [18]. Serum iron concentrations may be lower during the systemic inflammatory response, which can confound interpretation, resulting in patients receiving unnecessary perioperative treatment to correct a deficiency that may not be present. Therefore, the presence of iron deficiency may be much lower if the systemic inflammatory response is considered.

Attempts have been made through various means to correct for this mathematically and using various measures of the systemic inflammatory response. We have previously proposed serum iron correction using acute-phase reactants C-reactive protein (CRP) and albumin, which are readily available in most patients undergoing surgery [13].

Iron deficiency anemia, characterized by hypochromic microcytic indices and depleted iron stores, accounts for up to 30% of anemia in patients presenting for elective surgery [14]. A larger proportion of patients demonstrate normocytic anemia, which is associated with elevated markers of systemic inflammation [15,16]. For these patients, inflammatory cytokines induce hepcidin expression, leading to sequestration of iron within the reticuloendothelial system and reduced iron availability for erythropoiesis, despite adequate or even elevated iron stores [17,18]. This state is known as functional iron deficiency and is a hallmark of anemia of chronic disease. Distinguishing true iron deficiency from inflammation-induced changes is clinically important, as misclassification may expose patients to unnecessary treatment or obscure the need for intervention.

Assessment of iron status in the perioperative period is complicated by the systemic inflammatory response triggered by surgical trauma. Acute-phase reactants such as C-reactive protein (CRP) and ferritin increase following surgery, whereas negative acute-phase reactants, such as albumin and transferrin, decrease [17,18]. Serum iron and transferrin saturation (TSAT) are two markers that are used to guide iron therapy and are suppressed in the early postoperative period. This may lead to overdiagnosis of iron deficiency anemia and unnecessary iron supplementation [18,21]. While a TSAT less than 20% is widely accepted as indicative of functional iron deficiency in the presence of normal ferritin [18], both serum iron and transferrin are influenced by inflammation and can confound interpretation.

The aim of this study was to apply this regression-based correction to patients undergoing elective total hip arthroplasty; to examine changes in serum, iron, transferrin, and TSAT during the postoperative systemic inflammatory response (SIR); and to assess whether adjusting these markers for inflammation reduces misclassification of iron deficiency. We hypothesized that systemic inflammation would systemically perturb iron homeostasis following surgery and that correcting for this effect would result in a more accurate assessment of iron status in the perioperative and convalescent periods.

## 2. Materials and Methods

### 2.1. Patient Study and Design

This was a prospective observational cohort study conducted at Glasgow Royal Infirmary, United Kingdom. Twenty adult patients (11 males and 9 females) aged 36–76 years (median, 58 years) undergoing elective primary THA were enrolled. The biochemical data analyzed in this study were obtained from a local prospective cohort primarily designed to investigate perioperative changes in iron-related and trace-element biomarkers following elective total hip arthroplasty. Serum iron, transferrin, C-reactive protein (CRP), and albumin were therefore included as part of the predefined analytical panel. Ferritin was not measured in this cohort and was not available for analysis.

Inclusion criteria required the absence of preoperative systemic inflammation, defined as a CRP concentration less than 6 mg/L. Patients with active infection, inflammatory disease, malignancy, or blood transfusions within the last 6 months were excluded.

This study was approved by the West of Scotland Research Ethics Committee, and written informed consent was obtained from all participants prior to inclusion. All procedures were conducted in accordance with the Declaration of Helsinki and institutional guidelines.

### 2.2. Blood Sampling and Biochemical Analysis

Peripheral venous blood samples were collected in EDTA tubes at five time points: preoperatively (baseline) and on postoperative days (PODs) 1, 2, 3, and 90. These intervals were selected to capture the early postoperative SIR and the later convalescent phase based on previous studies examining perioperative inflammatory kinetics [24,25].

All biochemical analyses were performed by the Department of Clinical Biochemistry, Glasgow Royal Infirmary. Serum iron was measured using a ferene-based colorimetric method, and albumin was measured using a bromocresol green dye-binding assay. CRP and transferrin were quantified using turbidimetric immunoassays. TSAT was calculated using the standard formula: $TSAT\;\left(\%\right)=\;\frac{Serum\;iron\;(µmol/L)\;\times \;100}{25\;\times \;Transferrin\;(g/L)}$. Where measured values fell below the limit of detection (LOD), the LOD value was used in calculations. All measurements were taken on an Advia 1650 auto-analyzer (Bayer Corporation, Tarrytown, NY, USA): serum total iron was measured chemically using ferene, albumin was measured by a BCG dye-binding method, and transferrin and C-reactive protein were measured using a turbidimetric assay after binding to a specific antibody. For CRP, the limit of detection was 5 mg/L. The inter-assay coefficient of variation was less than 3% and 5% over the sample concentration range for albumin and CRP, respectively. There were no sustained concerns regarding IQC performance requiring investigation into the performance of the assays. The A, B, and C scores were within the EQA (NEQAS) targets during the study period.

### 2.3. Adjustment for Systemic Inflammation

Serum iron concentrations were adjusted for the effects of systemic inflammation using a previously validated linear regression equation developed by McSorley et al. [21]. This equation incorporates observed CRP and albumin (*Alb*) values and corrects the serum iron concentration based on reference values derived from a large cohort of preoperative patients:

$$Iron_{adj}=Iron_{obs}+\;\beta_{1}\left(CRP_{ref}-CRP_{obs}\right)+\;\beta_{2}\left(Alb_{obs}-Alb_{ref}\right)$$

where $Iron_{obs}\;$, $CRP_{obs}$, and $Alb_{obs}$ are observed concentrations and $CRP_{ref}$ and $Alb_{ref}$ are reference values derived from a cohort of 7226 patients [21]. Regression coefficients ($\beta_{1}$ and $\beta_{2}$) were obtained from the same model [21]. Adjusted transferrin saturation ($TSAT_{adj}$) was calculated using the adjusted serum iron concentration.

### 2.4. Definitions of Iron Deficiency

Standard definitions of iron deficiency were applied. Absolute iron deficiency was defined as serum iron less than 10 µmol/L, and functional iron deficiency was defined as TSAT less than 20%. Adjusted iron and TSAT values were compared to unadjusted measurements at each postoperative time point to assess their effect on the classification of iron deficiency.

### 2.5. Statistical Analysis

All analyses were performed using IBM SPSS Statistics version 26 (IBM Corp., Armonk, NY, USA). Continuous variables were expressed as medians (ranges) and compared across time using non-parametric repeated-measures ANOVA. Wilcoxon’s signed-rank test was used for paired comparisons between preoperative and postoperative values. Categorical variables, including the proportion of patients classified as iron-deficient, were compared using McNemar’s test. A two-tailed *p*-value of less than 0.05 was considered statistically significant.

## 3. Results

### 3.1. Patient Characteristics

Twenty patients (11 men and 9 women) were included. All patients had no biochemical evidence of systemic inflammation preoperatively. The median baselines for all the measurements were within our laboratory-derived population reference ranges (Table 1). No patients required allogenic blood transfusion, and no postoperative complications were recorded within the 90-day follow-up period (Table 1).

### 3.2. Systemic Inflammatory Response Following Arthroplasty

**Figure 1:**
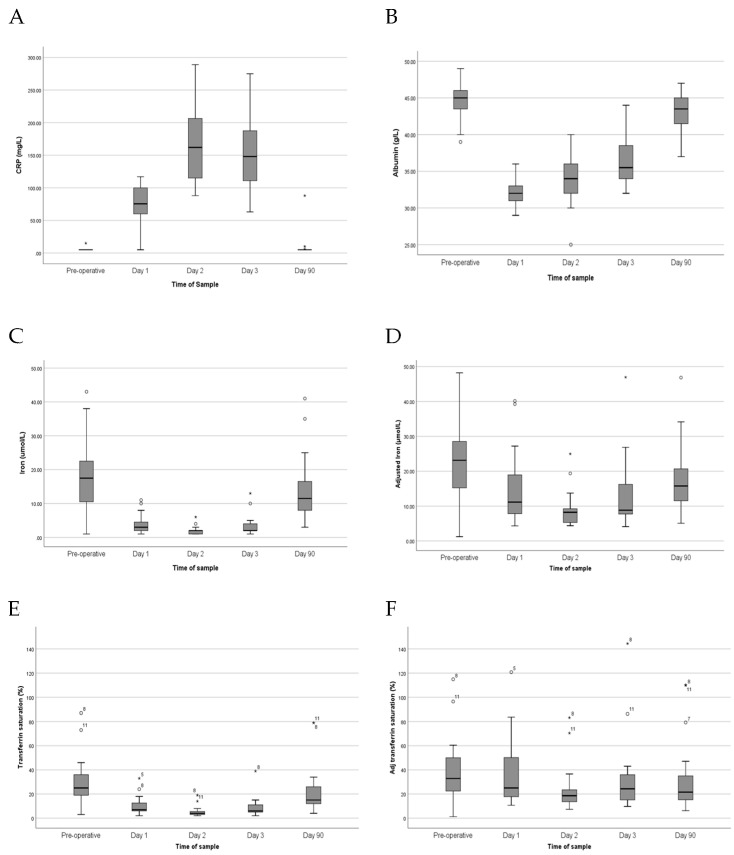
Boxplot of C-reactive protein concentrations (**A**), albumin concentrations (**B**), serum iron concentrations (**C**), serum iron concentrations adjusted for C-reactive protein and albumin (**D**), transferrin saturation (**E**), and transferrin saturation using serum iron adjusted for C-reactive protein and albumin (**F**) at each sampling time point. * and ^o^ represent outliers.

A marked systemic inflammatory response followed surgery (Figure 1). Median CRP increased from <6 mg/L at baseline to 83 mg/L on POD1 and peaked at 162 mg/L (92–289 mg/L) on POD 2 (*p* < 0.001, Table 2). By POD 90, CRP values returned to baseline. Albumin demonstrated an acute-phase fall, decreasing from a preoperative median of 45 g/L to 32 g/L on POD 1 (*p* < 0.0001, Table 2), and remained slightly reduced at POD 90 (44 g/L vs. 45 g/L preoperatively, *p* = 0.004, Table 2).

### 3.3. Changes in Iron Parameters

Iron indices showed profound suppression in the early postoperative period. Serum iron levels declined by almost 90% on POD 2 (17.5 µmol/L to 2.0 µmol/L, *p* < 0.001; Table 2). Although levels improved by day 90, they remained significantly lower than the baseline (11.5 µmol/L compared to 17.5 µmol/, *p* = 0.038; Table 2). Transferrin saturation showed a parallel decline, falling from 24% preoperatively to 4% on POD 2 (*p* < 0.001) and remained reduced, at 15%, on POD 90 (*p* = 0.015, Table 2). Transferrin concentrations also decreased significantly from 2.6 g/L to 1.5 g/L on POD 1 (*p* < 0.001, Table 2) but had returned to preoperative levels by day 90 (*p* = 0.600, Table 2).

Application of the regression-based correction for CRP and albumin attenuated the apparent postoperative decline in iron status. On POD 2, the adjusted median serum iron was 8.2 µmol/L, compared with 2.0 µmol/L unadjusted (*p* < 0.001). Adjusting transferrin saturation resulted in a similar pattern, with values of 19% versus 4% unadjusted (*p* < 0.001, Table 2). By day 90, adjusted and unadjusted measurements had converged, consistent with resolution of inflammation.

### 3.4. Reclassification of Iron Deficiency After Adjustment

Adjustment of iron indices for systemic inflammation resulted in substantial reclassification of iron deficiency status in the early postoperative period. Using conventional unadjusted thresholds, nearly all patients were classified as iron-deficient in the first three postoperative days (Table 3 and Table 4). After adjustment, the proportion of patients meeting criteria for iron deficiency was significantly reduced at each postoperative time point.

Using the conventional threshold of serum iron <10 µmol/L, 90% [18/20] of patients would have been categorized as iron-deficient on POD 1, and all the patients would have been classified as such on POD 2. With adjustment, these figures fell to 45% and 80%, respectively. By POD 3, 90% were iron-deficient based on unadjusted measures, compared with 60% after adjustment (*p* = 0.031, Table 3). A similar pattern was observed for transferrin saturation: 90% of patients were classified as iron-deficient on POD 1, and 100% were classified as such on POD 2 using unadjusted values, compared to 30% and 55% after adjustment (both *p* < 0.01, Table 4). On POD 3, adjustment of serum iron reduced the prevalence of iron deficiency from 90% (18/20) to 60% (12/20), corresponding to reclassification of six patients. On POD 90, unadjusted measurements suggested iron deficiency in 40% of patients by serum iron measurement and 60% by transferrin saturation, whereas adjustment reduced these estimates to 15% and 35% (*p* = 0.063, Table 3 and Table 4). Using transferrin saturation, adjustment reduced the prevalence of functional iron deficiency on POD 3 from 95% (19/20) to 35% (7/20), reclassifying twelve patients. Notably, the patients who remained classified as iron-deficient after adjustment on postoperative day 3 were the same individuals identified on postoperative day 90 (Table 4), suggesting that inflammation-adjusted indices preferentially identify patients with persistent iron deficiency rather than transient postoperative suppression.

Five patients moved from the ‘iron-deficient’ classification to ‘not iron deficient’ after using adjusted iron (8 to 3 patients; Table 3) and TSAT (12 to 7 patients; Table 4) measurements. Additionally, the iron-deficient patients, defined as serum iron < 10 µmol/L (n = 3; Table 3) were a subset of iron-deficient patients, defined as TSAT < 20% (n = 7; Table 4).

## 4. Discussion

This study demonstrates that systemic inflammation following total hip arthroplasty leads to a marked suppression of serum iron, transferrin, and transferrin saturation. As a result, nearly all patients met biochemical criteria for iron deficiency in the first three postoperative days. Adjustment of iron indices for CRP and albumin substantially reduced this apparent prevalence, halving the number of patients categorized as iron-deficient, and by three months, adjusted values no longer differed from preoperative baselines. These findings highlight the limitations of standard iron indices in the perioperative setting and suggest that adjustment for inflammation provides a more accurate representation of true iron status.

The clinical implications of these observations are significant. Preoperative anemia and iron deficiency are common among patients undergoing arthroplasty and are associated with increased transfusion requirements, higher complication rates, and poorer outcomes [1,2,3,6]. Current perioperative pathways frequently include routine iron studies, and intravenous iron supplementation is widely used to optimize patients before and after surgery [2,7,26]. However, our findings indicate that conventional iron indices are profoundly altered by surgical inflammation, leading to an overestimation of iron deficiency in the early postoperative period. If used uncritically, these indices may prompt unnecessary iron supplementation or investigations while others who would benefit most are not clearly distinguished. This comes with implications for cost, patient safety, and clinical decision-making [4,5,7,12,18,27].

Recent literature underscores the importance of this refinement. Park et al., in the largest systematic review and meta-analysis to date, demonstrated that perioperative intravenous iron reduces transfusion requirements and supports hemoglobin recovery after hip and knee arthroplasty, particularly when higher-dose regimens are used [23]. These findings establish the therapeutic value of IV iron in arthroplasty. Similarly, Varghese et al. showed that universal pre-operative iron studies with targeted IV iron supplementation were cost-neutral, associated with a trend toward lower transfusion rates, and offered the additional benefit of detecting occult disease [26]. The present work complements these findings by demonstrating that conventional iron indices are unreliable in the immediate postoperative period due to inflammation-related suppression. From a practical perspective, inflammation-adjusted iron indices could be readily integrated into existing perioperative anemia pathways without substantial additional resource requirements. C-reactive protein and albumin are already routinely measured in many perioperative settings, and the proposed correction could be applied automatically to serum iron and transferrin saturation using simple regression-based algorithms embedded within laboratory reporting systems or electronic health records (EHRs). Such integration could allow clinicians to distinguish transient postoperative inflammatory suppression of iron indices from true or persistent iron deficiency at the point of care, thereby reducing inappropriate investigation or treatment. Incorporation of this approach into EHR-based clinical decision support tools or automated alerts could further enhance its utility, flagging patients with adjusted indices suggestive of persistent iron deficiency for targeted review or intervention. This may be particularly relevant in enhanced recovery pathways, where early postoperative decision-making regarding iron supplementation, transfusion avoidance, and discharge planning is common.

In addition, this framework could complement emerging point-of-care testing strategies by providing an interpretive layer that accounts for systemic inflammation rather than relying on unadjusted thresholds. While point-of-care iron testing alone may be vulnerable to the same inflammatory confounding observed in standard laboratory assays, pairing such measurements with concurrent inflammatory markers and automated adjustment may improve diagnostic specificity. By applying an adjustment for CRP and albumin, iron status can be assessed more accurately, allowing for better identification of patients who are truly iron-deficient and most likely to benefit from supplementation. The present findings suggest a practical role for inflammation-adjusted indices in perioperative pathways. Correction for inflammation may distinguish true iron deficiency from transient inflammatory suppression, improving selection of patients for supplementation. Future studies should evaluate whether integration of inflammation-adjusted iron indices into perioperative care pathways improves patient selection for intravenous iron therapy and translates into measurable benefits in transfusion rates, recovery trajectories, and healthcare resource utilization.

Several limitations should be acknowledged. This was a small, single-center pilot study, and the findings require validation in larger, more diverse cohorts. Ferritin and red-cell indices were not included in this analysis, although these are, themselves, influenced by inflammation and may not have altered the principal conclusions. The correction model was derived from a non-arthroplasty cohort and requires prospective validation specifically in orthopedic surgery populations. Ferritin was not available in this cohort, as the study was nested within a broader trace-element analysis; however, given its behavior as a positive acute-phase reactant, inclusion of ferritin would not have materially altered the interpretation of iron status in the early postoperative period. Finally, we did not assess patient-centered outcomes such as functional recovery or quality of life, which remain important endpoints for future research.

## 5. Conclusions

Systemic inflammation following total hip arthroplasty profoundly suppresses serum iron and transferrin saturation, leading to widespread misclassification of iron deficiency when conventional indices are applied. Correcting these measures for CRP and albumin substantially reduces misclassification and provides a more accurate assessment of perioperative iron status. Placed alongside strong evidence for the efficacy of intravenous iron in arthroplasty, these findings highlight the importance of reliable diagnostics to guide therapy. Inflammation-adjusted indices represent a practical refinement that could help target supplementation to the patients most likely to benefit and should be validated in larger, multicenter studies.

## Figures and Tables

**Table 1:** Characteristics and baseline measurements.

Characteristic	Reference Interval	Operative Cohort (n = 20)
Age (years)	NA	58 (35–76)
Sex (male/female)	NA	11 (55%)/9 (45%)
C-reactive protein (mg/L)	<6	<6 (<6–<6)
Albumin (g/L)	35–55	45 (39–49)
Serum Iron (µmol/L)	10–30	17.5 (1.0–43.0)
Transferrin (g/L)	2.0–3.5	2.6 (1.9–4.0)
Transferrin saturation (%)	25–45%	24 (3–73)

Continuous variables expressed as medians and ranges.

**Table 2:** Plasma concentrations (baseline and postoperative days 1, 2, 3, and 90) of CRP, albumin, serum iron, adjusted serum iron, and transferrin, as well as transferrin saturation, following elective total hip replacement.

	Albumin (g/L)	CRP (mg/L)	Iron (µmol/L)	Adj. Iron (µmol/L)	Transferrin (g/L)	TSAT (%)	Adj TSAT (%)
**Pre–operative**	45 (39–49)	<6 (<6–<6)	17.5 (1.0–43.0)	23.1 (1.2–54.6)	2.6 (1.9–4.0)	25 (3–87)	33 (1–115)
**POD 1**	32 (29–36)	83 (35–117)	3.0 (1.0–11.0)	11.1 (4.3–40.1)	1.5 (1.0–2.8)	7 (2–33)	25 (11–121)
**POD 2**	34 (25–40)	162 (92–289)	2.0 (1.0–6.0)	8.2 (4.4–25.0)	1.6 (1.1–2.8)	4 (1–39)	19 (7–83)
**POD 3**	36 (32–44)	148 (63–275)	2.0 (1.0–13.0)	8.8 (4.1–46.9)	1.6 (1.0–2.8)	6 (2–39)	24 (10–144)
**POD 90**	44 (37–47)	<6 (<6–88)	11.5 (3.0–41.0)	15.8 (5.1–54.9)	2.9 (1.5–4.1)	15 (4–79)	22 (6–110)
***p*** **-values**							
ANOVA **^1^**	<0.001	<0.001	<0.001	<0.001	<0.001	<0.001	0.097
Pre-op/POD1 **^2^**	<0.001	<0.001	<0.001	0.028	<0.001	0.001	0.478
Pre-op/POD2 **^2^**	<0.001	<0.001	<0.001	<0.001	<0.001	0.001	0.002
Pre-op/POD90 **^2^**	0.004	0.109	0.038	0.079	0.600	0.015	0.204

Continuous variables are presented as medians and ranges. ^1^ *p*-value derived from repeated-measures ANOVA. ^2^ *p*-value derived from Wilcoxon’s signed-rank test comparing differences between preop and day-2 measurements and between preop and day-90 measurements.

**Table 3:** Comparison of serum iron measurements with measurements adjusted for CRP and albumin when estimating the prevalence of iron deficiency, defined as serum iron < 10 µmol/L, in a cohort of total hip replacement patients.

	Unadjusted Iron Measurement	Adjusted Iron Measurement	*p*-Value
**Serum iron (µmol/L)**			
Preoperative	17.5 (1.0–43.0)	23.1 (1.2–54.6)	<0.001 **^1^**
Day 1	3.0 (1.0–11.0)	11.1 (4.3–40.1)	<0.001 **^1^**
Day 2	2.0 (1.0–6.0)	8.2 (4.4–25.0)	<0.001 **^1^**
Day 3	2.0 (1.0–13.0)	8.8 (4.1–46.9)	<0.001 **^1^**
Day 90	11.5 (3.0–41.0)	15.8 (5.1–54.9)	<0.001 **^1^**
**Iron < 10 µmol/L, n (%)**			
Preoperative	3 (15)	3 (15)	1.000 **^2^**
Day 1	18 (90)	9 (45)	0.004 **^2^**
Day 2	20 (100)	16 (80)	0.125 **^2^**
Day 3	18 (90)	12 (60)	0.031 **^2^**
Day 90	8 (40)	3 (15)	0.063 **^2^**

Continuous variables are presented as medians and ranges. ^1^ *p*-value derived from Wilcoxon’s signed-rank test comparing differences between adjusted iron and non-adjusted serum iron measurements. ^2^ *p*-value calculated using McNemar’s test to compare the difference between the proportions of iron-deficient patients between adjusted and non-adjusted serum iron measurements.

**Table 4:** Comparison of transferrin saturation (TSAT) measurements with TSAT measurements, where serum iron is adjusted for CRP and albumin when estimating the prevalence of normocytic functional iron deficiency anemia, defined as TSAT < 20%, in a cohort of total hip replacement patients.

	Unadjusted Iron TSAT Measurement	Adjusted Iron TSAT Measurement	*p*-Value
**TSAT (%)**			
Preoperative	25 (3–87)	33 (1–115)	<0.001 **^1^**
Day 1	7 (2–33)	25 (11–121)	<0.001 **^1^**
Day 2	4 (1–39)	19 (7–83)	<0.001 **^1^**
Day 3	6 (2–39)	24 (10–144)	<0.001 **^1^**
Day 90	15 (4–79)	22 (6–110)	<0.001 **^1^**
**TSAT < 20%, n (%)**			
Preoperative	5 (25)	3 (15)	0.625 **^2^**
Day 1	18 (90)	6 (30)	<0.001 **^2^**
Day 2	20 (100)	11 (55)	0.004 **^2^**
Day 3	19 (95)	7 (35)	<0.001 **^2^**
Day 90	12 (60)	7 (35)	0.063 **^2^**

Continuous variables are presented as medians and ranges. ^1^ *p*-value derived from Wilcoxon’s signed-rank test comparing differences between adjusted iron and non-adjusted serum iron measurements. ^2^ *p*-value is calculated using McNemar’s test to compare the difference between the proportions of iron-deficient patients between adjusted and non-adjusted serum iron measurements.

## Data Availability

The raw data supporting the conclusions of this article will be made available by the authors upon request.

## Abbreviations

The following abbreviations are used in this manuscript:

AGP	α-1-acid glycoprotein
BRINDA	Biomarkers Reflecting Inflammation and Nutritional Determinants of Anemia
CRP	C-reactive protein
EPO	Erythropoietin
Hb	Hemoglobin
LOD	Limit of detection
MCH	Mean corpuscular hemoglobin
MCV	Mean corpuscular volume
RBC	Red blood cell count
TSAT	Transferrin saturation
